# Ectromelia Virus Affects Mitochondrial Network Morphology, Distribution, and Physiology in Murine Fibroblasts and Macrophage Cell Line

**DOI:** 10.3390/v10050266

**Published:** 2018-05-16

**Authors:** Karolina P. Gregorczyk, Zbigniew Wyżewski, Joanna Szczepanowska, Felix N. Toka, Matylda B. Mielcarska, Magdalena Bossowska-Nowicka, Małgorzata Gieryńska, Anna Boratyńska-Jasińska, Justyna Struzik, Marek G. Niemiałtowski, Lidia Szulc-Dąbrowska

**Affiliations:** 1 Division of Immunology, Department of Preclinical Sciences, Faculty of Veterinary Medicine, Warsaw University of Life Sciences—SGGW, Ciszewskiego 8, 02-786 Warsaw, Poland; karolina_gregorczyk@sggw.pl (K.P.G.); zbigniew_wyzewski@sggw.pl (Z.W.); FToka@rossvet.edu.kn (F.N.T.); matyldan@gmail.com (M.B.M.); bossowska.magdalena@gmail.com (M.B.-N.); malgorzata_gierynska@sggw.pl (M.G.); justyna_struzik@sggw.pl (J.S.); marek_niemialtowski@sggw.pl (M.G.N.); 2 Laboratory of Bioenergetics and Biomembranes, Department of Biochemistry, Nencki Institute of Experimental Biology, Polish Academy of Science, Pasteura 3, 02-093 Warsaw, Poland; j.szczepanowska@nencki.gov.pl; 3 Integrative and Mammalian Research Centre, Department of Biomedical Sciences, Ross University School of Veterinary Medicine, P.O. Box 334 Basseterre, Saint Kitts and Nevis; 4 Molecular Biology Unit, Mossakowski Medical Research Centre, Polish Academy of Sciences, Pawińskiego 5, 02-106 Warsaw, Poland; aboratynska@imdik.pan.pl

**Keywords:** mitochondrial network, ectromelia virus, mitochondrial membrane potential, reactive oxygen species, mitochondrial mass

## Abstract

Mitochondria are multifunctional organelles that participate in numerous processes in response to viral infection, but they are also a target for viruses. The aim of this study was to define subcellular events leading to alterations in mitochondrial morphology and function during infection with ectromelia virus (ECTV). We used two different cell lines and a combination of immunofluorescence techniques, confocal and electron microscopy, and flow cytometry to address subcellular changes following infection. Early in infection of L929 fibroblasts and RAW 264.7 macrophages, mitochondria gathered around viral factories. Later, the mitochondrial network became fragmented, forming punctate mitochondria that co-localized with the progeny virions. ECTV-co-localized mitochondria associated with the cytoskeleton components. Mitochondrial membrane potential, mitochondrial fission–fusion, mitochondrial mass, and generation of reactive oxygen species (ROS) were severely altered later in ECTV infection leading to damage of mitochondria. These results suggest an important role of mitochondria in supplying energy for virus replication and morphogenesis. Presumably, mitochondria participate in transport of viral particles inside and outside of the cell and/or they are a source of membranes for viral envelope formation. We speculate that the observed changes in the mitochondrial network organization and physiology in ECTV-infected cells provide suitable conditions for viral replication and morphogenesis.

## 1. Introduction

Mitochondria are multifunctional organelles in eukaryotic cells. In addition to their primary role in ATP production through oxidative phosphorylation, they play a key role in numerous crucial cellular processes, including buffering cytosolic calcium ions, fatty acid oxidation, and metabolism of amino acids and lipids, as well as cell proliferation and cell death [1,2]. Regarding their role in apoptosis, mitochondria regulate the removal of virus-infected cells, thus becoming part of the innate antiviral immunity. Moreover, recent studies on mitochondrial functions have indicated that these organelles emerge as a fundamental platform for innate immune signaling against viral infection via mitochondrial antiviral signaling protein (MAVS) [3], also known as VISA (virus-induced signaling adaptor) [4], IPS1 (IFNβ promoter stimulator 1) [5], or Cardif (CARD adaptor inducing IFNβ) [6]. MAVS is associated with the outer mitochondrial membrane (OMM) and transduces downstream signaling that culminates in the production of type I interferons (IFNs) and pro-inflammatory cytokines upon RIG-like receptors (RLRs, RIG-I—retinoic acid-inducible gene I, MDA-5—melanoma differentiation-associated protein 5) and the binding of pathogen-associated molecular patterns such as dsRNA or ssRNA during infection [7]. However, many types of viruses have evolved mechanisms to evade the mitochondrial-mediated antiviral innate immune response via modulation of an intrinsic apoptotic pathway [8] and by blocking the production of antiviral proteins [9,10,11].

Functions of mitochondria depend on their distribution inside the cell and morphology of their network that is determined by the balance between fusion and fission events of the outer and inner mitochondrial membranes [12]. Organelle fission is mediated by recruitment of dynamin-related protein 1 (Drp1) to the OMM, where the formation of high-molecular-weight protein complexes marking fission sites occurs. Recruitment of Drp1 from the cytosol to mitochondria is mediated by mitochondrial fission factor (Mff), mitochondrial division 49/51 (MiD49/51), and, less apparently, fission protein 1 (Fis1), localized in the OMM [13]. However, it has been shown that both Fis1 and Mff have a role in mitochondrial fission and are important for the number and size of Drp1 puncta on mitochondria in MEFs [14]. Moreover, Fis1 and Mff can act independently of one another, and partially replace each other to regulate mitochondria fission. Further, MiD49 and MiD51 can mediate Drp1 recruitment and mitochondrial fission in the absence of Fis1 and Mff. These results demonstrate that mitochondrial fission can be mediated by multiple receptors, which are able to recruit Drp1 [14]. Mitochondrial fusion is a two-step process. First, OMM fusion occurs and is facilitated by mitofusin 1 (Mfn1) and mitofusin 2 (Mfn2). In the next step, optic atrophy protein 1 (Opa1) allows fusion of the inner membrane (IMM) [12], leading to the formation of elongated mitochondria. Imbalances in mitochondrial fusion and fission processes have been found in neurodegenerative (e.g., Parkinson disease, Alzheimer disease, Huntington disease) and autoimmune (e.g., multiple sclerosis, type 1 diabetes) diseases, as well as tumors [15]. Recent studies have shown that bacterial [16] and viral infections [17] also can affect mitochondrial dynamics.

In our study, we focused on ectromelia virus (ECTV), the causative agent of mousepox. The virus belongs to the *Poxviridae* family and *Orthopoxvirus* genus that also includes variola virus (VARV, the causative agent of smallpox) and vaccinia virus (VACV). ECTV is closely related to VARV because of the narrow host range and similar disease symptoms; however, it does not pose a risk to human health. Therefore, ECTV has been used repeatedly as a model for investigating pathogenesis of orthopoxvirus infections [18]. Many studies have revealed that orthopoxviruses affect mitochondrial-mediated apoptosis [19,20,21]. However, little is known about the impact of orthopoxviral infection on the morphology and physiology of mitochondria.

Poxviruses are distinguished from other DNA viruses, as their replication occurs exclusively in the cytoplasm of infected cells in foci known as viral factories. This unique feature requires intracellular reorganization of the cytoskeleton and organelles, including mitochondria, endoplasmic reticulum, lysosomes, endosomes, and Golgi apparatus [22]. Our previous study indicated that ECTV infection leads to cytoskeletal rearrangement and alterations in mitochondrial network morphology and distribution in established cell lines [23,24,25]. In the present study, we asked how ECTV infection affects mitochondrial network morphology and physiology in permissive cells. We selected nonimmune (fibroblasts) and immune (macrophages) cells that are present at the site of virus entry and participate in the pathogenesis of mousepox. Results revealed that in the early stages of infection (4 h post infection (hpi)), mitochondria began to accumulate near the viral factories, especially in the area between the nucleus and the viral factories. During later stages of infection (18 hpi), mitochondria had altered physiology, including a decrease in mitochondrial membrane potential and mitochondrial mass, imbalance between mitochondrial fission–fusion, and increase in generation of reactive oxygen species (ROS), suggesting damage to mitochondria. The results suggest that ECTV-induced changes in the mitochondrial network organization and physiology provide suitable conditions for viral replication and morphogenesis.

## 2. Materials and Methods

### 2.1. Virus and Cell Lines

Highly virulent Moscow strain ECTV (ECTV-MOS; ATCC VR1374) was propagated and titrated by plaque assay (PFU/mL) in Vero cell culture (African green monkey kidney epithelial cells; ATCC CCL-81). The virus was purified and stored at −70 °C until use.

L929 murine fibroblasts (ATCC CCL1) were maintained according to the protocol previously described in [25]. The murine macrophages RAW 264.7 (ATCC TIB-71) were cultured in RPMI 1640-GlutaMAX medium (Gibco, Waltham, MA, USA) supplemented with 10% FBS and 1% antibiotic solution containing 100 U/mL penicillin and 100 µg/mL streptomycin (Sigma-Aldrich, Saint Louis, MO, USA) at 37 °C, with 5% CO_2_ in a humidified incubator. Wild-type murine embryonic fibroblasts (MEFs^WT^; ATCC CRL-2991) and Mfn1- and Mfn2-deficient MEFs (MEFs^Mfn1−/−/Mfn2−/−^) (ATCC CRL-2994) were maintained in DMEM with 4.5 g/L glucose and 4.0 mM l-glutamine supplemented with 10% FBS and 1% antibiotic–antimycotic solution containing 100 U/mL penicillin, 100 µg/mL streptomycin, and 0.25 µg/mL amphotericin B at 37 °C, with 5% CO_2_ in a humidified incubator. L929, RAW 264.7, MEFs^WT^, MEFs^Mfn1−/−/Mfn2−/−^ and Vero cell lines are permissive to ECTV infection.

### 2.2. Fluorescent Probes and Antibodies

Mito Red dye (Sigma-Aldrich) was used to visualize the mitochondrial network morphology and distribution. The mitochondrial mass was determined with the aid of MitoTracker Green FM (ThermoFisher Scientific, Waltham, MA, USA). Mitochondrial membrane potential was measured with the fluorescence probe JC-1 (5,5′,6,6′-tetrachloro-1,1′,3,3′-tetraethylbenzimidazol-carbocyanine iodide; ThermoFisher Scientific) and ROS were detected using CM-H_2_DCFDA (5-(6)-chloromethyl-2′,7′-dichlorodihydrofluorescein diacetate; ThermoFisher Scientific). Phalloidin-tetramethylrhodamine B isothiocyanate (TRITC) or phalloidin-fluorescein isothiocyanate (FITC; Sigma-Aldrich) was used to detect F-actin. DNA was visualized by labeling with Hoechst 33342 (Sigma-Aldrich). Other cellular structures including mitochondrial proteins were stained with the following primary antibodies (Abs): mouse monoclonal Abs (mAbs) anti-α-tubulin (Sigma-Aldrich); mouse mAbs anti-γ-tubulin (Sigma-Aldrich); mouse mAb anti-Drp1 (BD Biosciences, Franklin Lakes, NJ, USA); mouse mAb anti-Opa1 (BD Biosciences); rabbit pAb anti-Fis1 (ThermoFisher Scientific); mouse polyclonal Ab anti-GAPDH (ThermoFisher Scientific); rabbit mAb anti-LC3B (ThermoFisher Scientific); rabbit pAbs anti-LC3B (Cell Signaling Technology, Danvers, MA, USA). Rabbit pAbs anti-ECTV conjugated with FITC were used for detection of viral antigens and were obtained as previously described [23]. Donkey anti-mouse and anti-rabbit secondary Abs conjugated with FITC or rhodamine Red-X (Jackson ImmunoResearch Laboratories, West Grove, PA, USA) were used for immunofluorescent staining. For Western blot analysis, primary Abs were detected with goat secondary Abs conjugated with horseradish peroxidase (HRP) directed against mouse or rabbit IgG (Santa Cruz Biotechnology, Dallas, TX, USA). Tetramethylbenzidine (TMB, ThermoFisher Scientific), a colorimetric substrate, was used for visualization.

### 2.3. Cell Line Infection with ECTV

Twenty-four-hour cultures of L929, RAW 264.7, MEFs^WT^, or MEFs^Mfn1−/−/Mfn2−/−^ cells were infected with ECTV at a multiplicity of infection (MOI) of 5 in DMEM supplemented with 1% FBS and 1% antibiotic–antimycotic solution (for L929, MEFs^WT^, and MEFs^Mfn1−/−/Mfn2−/−^) or in RPMI 1640-GlutaMAX medium supplemented with 2% FBS and 1% antibiotic solution (for RAW 264.7). Uninfected (culture without ECTV) cells were used as a negative control. At 4, 8, 12, 18, and/or 24 hpi, cells were harvested for further analysis.

### 2.4. Plaque Assay

Vero cells were seeded in 24-well plates and infected with 10-fold serial dilutions of ECTV suspension obtained from RAW 264.7 macrophages at 4, 12, and 24 hpi. At 5 days post infection, Vero cell monolayers were fixed in 4% paraformaldehyde (PFA, Sigma-Aldrich), stained with 0.3% crystal violet, and air dried. Plaques were counted using an Olympus IX71 inverted microscope (Olympus, Tokyo, Japan).

### 2.5. Immunofluorescent Staining and Microscopy Analysis

Permissive cells were seeded on glass coverslips in a 24-well plate and mitochondria were labeled with 300 nM Mito Red (560/580 [Ex/Em]) for 20 min in the dark at 37 °C with 5% CO_2_ in a humidified incubator. The interaction of Mito Red with mitochondria depends on the mitochondrial membrane potential. After incubation, cells were washed three times with warm (37 °C) culture medium for 5 min. To detect ECTV antigens, F-actin, or Drp1, cells were fixed with 4% PFA in PBS for 20 min; for α-tubulin labeling, cells were fixed in ice-cold methanol (Sigma-Aldrich) for 2 min. Fixed cells were then permeabilized with 0.5% Triton X-100 (Sigma-Aldrich) in PBS (15 min) and blocked with 3% bovine serum albumin (BSA, Sigma-Aldrich) in 0.1% Triton X-100-PBS solution (30 min) to prevent nonspecific binding. The cells were subsequently incubated with primary Abs anti-Drp1 or α-tubulin for 1 h. Next, secondary anti-mouse Abs conjugated with FITC or rhodamine Red-X were used (60 min in the dark). Viral antigens were stained with FITC-conjugated rabbit anti-ECTV Abs, as previously described [24]. For F-actin detection, cells were incubated with 0.5 µg/mL phalloidin-FITC or -TRITC for 20 min in the dark. DNA was stained with 1 µg/mL Hoechst 33342 for 5 min in the dark. Slides were mounted in ProLong Gold Antifade Reagent (ThermoFisher Scientific).

Autophagy was detected with an LC3B Antibody Kit for Autophagy (ThermoFisher Scientific) containing anti-LC3B antibodies and chloroquine diphosphate. Before staining with Mito Red, the uninfected cells were pretreated with 30 µM chloroquine disphosphate for 16 h and used as a positive control. After fixation and permeabilization, the cells were incubated with primary anti-LC3B Abs for 1 h. Next, secondary anti-rabbit antibodies conjugated with FITC were used (1 h in the dark).

Images were recorded using an Olympus BX60 fluorescence microscope (Olympus) equipped with a Color View III cooled CCD camera, and confocal microscopes Leica SP8 (Leica, Wetzlar, Germany) and Zeiss LSM780 (Zeiss, Jena, Germany), and processed using the Cell^F (Olympus), CellSens Dimension (Olympus, version 1.8), and ImageJ (NIH Image, version 1.50i, Bethesda, MD, USA) software.

### 2.6. Transmission Electron Microscopy (TEM) Analysis

At 18 hpi, control and ECTV-infected L929 and RAW 264.7 cells grown in 24-well culture plates were collected and fixed for 2 h with 2.5% glutaraldehyde in phosphate buffer. After fixation, cells were rinsed in phosphate buffer and embedded in 2% agarose to form agarose blocks and then cut into smaller pieces. Next, cells were post-fixed for 1 h with 1% osmium tetroxide. Cells were dehydrated for 10 min at room temperature (RT) in each solution of ethanol with increasing concentrations (70% and 95%), twice in absolute ethanol for 10 min at RT and once in propylene oxide for 20 min at 4 °C. In the next step, cells were embedded in epoxy resin and cut into sections using a Leica EM UC6 ultramicrotome (Leica). The samples were examined with transmission electron microscope Jeol JEM-1220 (JEOL Ltd., Tokyo, Japan) equipped with an SIS Morada 11 MP camera.

### 2.7. Scanning Electron Microscopy (SEM) Analysis

At 18 hpi, control and ECTV-infected L929 and RAW 264.7 cells grown on glass coverslips in a 24-well plate were fixed for 1 h with 2.5% glutaraldehyde in phosphate buffer. Next, cells were rinsed in phosphate buffer and post-fixed for 1 h with 1% osmium tetroxide in phosphate buffer. Fixed cells were dehydrated for 10 min at RT in each solution of ethanol with increasing concentrations (70% and 95%), twice in absolute ethanol for 10 min at RT, and twice in acetone for 10 min at RT. Next, samples were dried using a CPD 7501 critical point drier (Polaron, Hatfield, PA, USA) and coated with a gold layer in a JFC-1300 sputter-coater (JEOL Ltd.). For analysis of specimens, the FEI Quanta 200 environmental scanning electron microscope (ESEM) with EDAX energy dispersive spectroscopy (EDS) system (FEI, Tokyo, Japan) was used.

### 2.8. Determination of Mitochondrial Membrane Potential

Mitochondrial membrane potential (MMP) was measured with a flow cytometer (LSRFortessa (BD Biosciences)) by using the dual-emission potential-sensitive probe JC-1 as a fluorescent dye. At high concentrations (due to high MMP), the dye aggregates, yielding red fluorescence with emission at ~590 nm, while at low concentration (due to low MMP), JC-1 is predominantly a monomer that yields green fluorescence with emission at ~529 nm. The ratio of red to green fluorescence of JC-1 is dependent only on MMP, and is not influenced by mitochondrial size, shape, or density. Therefore, mitochondrial depolarization is indicated by a decrease in the red/green fluorescence intensity ratio.

At indicated time points, control and ECTV-infected L929 and RAW 264.7 cells grown in 12-well culture plates were collected and incubated with culture medium supplemented with 10 or 6 µM JC-1 dye, respectively, for 20 min in the dark at 37 °C with 5% CO_2_ in a humidified incubator. Cells were then centrifuged, suspended in cold (4 °C) PBS, and analyzed by flow cytometry (λ_ex_ = 488 nm). Data were presented as the ratio of red to green fluorescence of JC-1 dye.

Uninfected L929 and RAW 264.7 cells treated both with JC-1 and 200 or 300 µM CCCP (carbonyl cyanide 3-chlorophenylhydrazone; Sigma-Aldrich), respectively, were used as negative control to confirm that the JC-1 response is sensitive to changes in MMP. CCCP is a protonophore (H^+^ ionophore) and uncoupler of oxidative phosphorylation in mitochondria that collapse MMP.

### 2.9. Measurement of Reactive Oxygen Species Level

Reactive oxygen species (ROS) levels were measured with the redox-sensitive dye CM-H_2_DCFDA by flow cytometry. Non-fluorescent CM-H_2_DCFDA is converted to highly fluorescent CM-DCF (5-(6)-chloromethyl-2′,7′-dichlorofluorescein) upon cleavage of the acetate groups by intracellular esterases and oxidation; [Ex/Em] ~492-495/517-527 nm. Control and ECTV-infected L929 and RAW 264.7 cells grown in 12-well culture plates were collected at indicated time points and incubated in PBS supplemented with 1 μM or 2 μM CM-H_2_DCFDA dye, respectively, for 10 min in the dark at 37 °C with 5% CO_2_ in a humidified incubator. Next, cells were centrifuged, suspended in cold (4 °C) PBS, and analyzed in a flow cytometer (λ_ex_ = 488 nm). Uninfected L929 and RAW 264.7 cells treated with both CM-H_2_DCFDA and 1 mM H_2_O_2_ were used as a positive control.

### 2.10. Measurement of Mitochondrial Mass

Mitochondrial mass was measured by flow cytometery (BD LSRFortessa, BD Biosciences) by using MitoTracker Green FM (490/516 nm [Em/Ex]). MitoTracker Green FM is a non-fluorescent dye in aqueous solutions, but becomes fluorescent once it accumulates in the lipid environment of mitochondria, regardless of membrane potential. At indicated time points, control and ECTV-infected L929 and RAW 264.7 cells prepared as described in Section 2.3, were collected and incubated with culture medium supplemented with 200 nM MitoTracker Green FM for 20 min in the dark at 37 °C with 5% CO_2_ in a humidified incubator. Cells were then centrifuged, suspended in cold (4 °C) PBS, and analyzed by flow cytometry (λ_ex_ = 488 nm).

### 2.11. Determination of ATP Levels

ATP levels were measured spectrophotometrically using an ATP Assay Kit (Abcam, Cambridge, MA, USA) which is based on the phosphorylation of glycerol in order to generate a product that can be easily quantified colorimetrically. At 18 hpi, control and ECTV-infected L929 cells (see Section 2.3) were collected and treated according to specifications in the ATP Assay Kit instruction manual. The absorbance of samples was measured using an Epoch Microplate Spectrophotometer (BioTek, Winooski, VT, USA) at OD 570 nm and the results were computed from the standard curve.

### 2.12. Western Blot Analysis

Control and ECTV-infected L929 and RAW 264.7 cells were lysed using RIPA buffer (ThermoFisher Scientific) supplemented with 1% of protease and phosphatase inhibitor cocktail (ThermoFisher Scientific). The protein concentrations were measured with a bicinchoninic acid (BCA) assay (ThermoFisher Scientific) according to the manufacturer’s instructions. Next, 10–20 μg of total lysate was separated with 10% sodium dodecyl sulfate polyacrylamide gel electrophoresis (SDS-PAGE) and transferred onto a polyvinylidene fluoride (PVDF) membrane. After blocking with 5% non-fat dry milk in 0.1% Tween 20-PBS solution, membranes were incubated with primary Abs overnight at 4 °C as follows: anti-Opa1, anti-Drp1, anti-LC3B—1:1000 dilution, anti-GAPDH—1:1000 dilution, or anti-Fis1—1:3000 dilution. Secondary Abs conjugated with HRP (1:10,000) were used. The membranes were developed using tetramethylbenzidine (TMB) or SuperSignal West Pico PLUS (Thermo Fisher Scientific) as substrates. Next, the results were visualized in the Molecular Imager VersaDoc system (Bio-Rad, Hercules, MA, USA) or using X-ray film. Glyceraldehyde 3-phosphate dehydrogenase (GAPDH) was used as a loading control and for protein normalization during densitometry measurements. ImageJ software was used for densitometry analysis.

### 2.13. Apoptosis Measurement by Flow Cytometry

The apoptotic rate of L929 and RAW 264.7 cells was determined using an Annexin V-FITC detection kit (Sigma-Aldrich) according to the manufacturer’s protocol. At indicated time points, control and ECTV-infected L929 and RAW 264.7 cells grown in 12-well culture plates were collected, washed twice with cold PBS, and resuspended in 1× binding buffer. In the next step, 1 × 10^5^ cells contained in 100 μL of binding buffer were labeled with 5 μL Annexin V-FITC and 5 μL propidium iodide (PI) and incubated for 15 min at RT in the dark. Then, cells were resuspended in 400 μL of 1× binding buffer and analyzed by flow cytometry (λ_ex_ = 488 nm).

The kit includes Annexin V conjugated with FITC to label phosphatidylserine sites on the membrane surface, and PI to label the cellular DNA in necrotic cells where the cell membrane has been totally compromised. It can differentiate among early apoptotic cells (annexin V positive, PI negative), late apoptotic or necrotic cells (annexin V positive, PI positive), and viable cells (annexin V negative, PI negative).

### 2.14. Statistical Analysis

The results were presented as means ± SD of at least three independent experiments. The significance of statistical differences was evaluated using Student’s *t*-test. The results were accepted as significantly different when *p* ≤ 0.05 *, *p* ≤ 0.01 **, or *p* ≤ 0.001 ***.

## 3. Results

### 3.1. Kinetics of ECTV Replication in Murine Cell Lines

**Figure 1:**
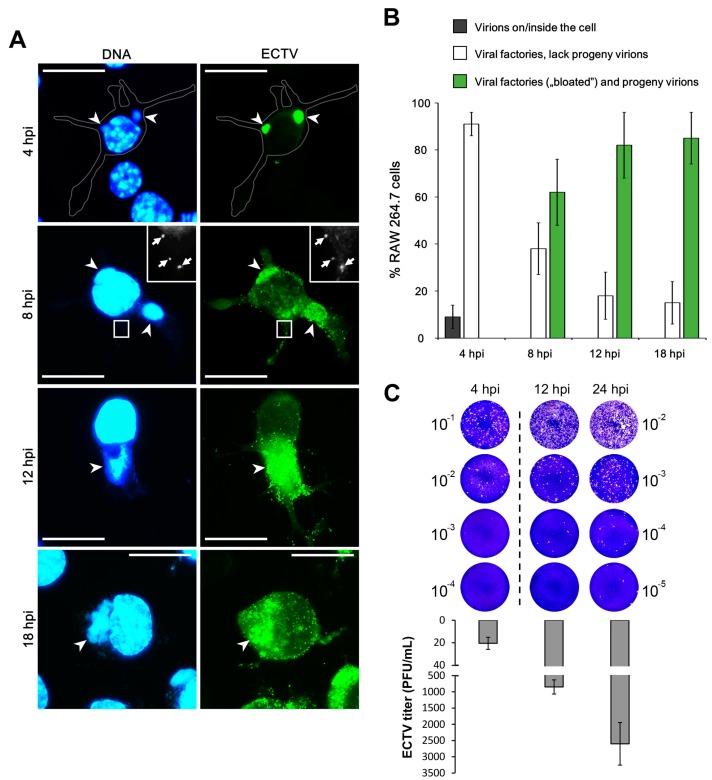
Replication of ectromelia virus (ECTV) in RAW 264.7 cells. (**A**) Arrowheads indicate the viral factories. Arrows indicate progeny virions. Scale bar: 10 µm. (**B**) Percentage of ECTV-infected cells with virions on/inside the cell, cells with viral factories without progeny virions, and cells with “bloated” viral factories and progeny virions (*n* = 100 cells). (**C**) ECTV titer in RAW 264.7 cells at 4, 12, and 24 hours post infection (hpi) determined by plaque assay. Plaques were visualized by staining infected Vero cell monolayers with 0.3% crystal violet. Data from three independent experiments are presented as mean ± SD. PFU—plaque forming unit.

The kinetics of ECTV replication in RAW 264.7 macrophages and L929 fibroblasts were determined by standard plaque assay and fluorescence microscopy visualization of ECTV antigens using rabbit polyclonal antibodies anti-ECTV conjugated with FITC. Viral and cellular (nuclear) DNA were labeled with Hoechst 33342. The first ECTV replication centers were observed at 4 hpi in RAW 264.7 cells, and this was determined as the early stage of infection (Figure 1A). Our recent studies have shown that viral factories in L929 fibroblasts also appeared at 4 hpi, but only 17% of cells displayed the presence of replication centers [25] in contrast to RAW 264.7 macrophages where 90% of ECTV-infected cells had viral replication centers (Figure 1B). Early viral factories were observed as regular shapes (round or oval), but at 8 hpi, 60% of RAW 264.7 cells presented irregular “bloated” viral factories with progeny virions, whereas L929 exhibited a similar stage not earlier than 12 hpi (Figure 1) [25]. Plaque assay confirmed that during ECTV infection in RAW 264.7 macrophages the number of infectious particles increased between 4 and 24 hpi (Figure 1C).

### 3.2. Cell Morphology during ECTV Infection

**Figure 2:**
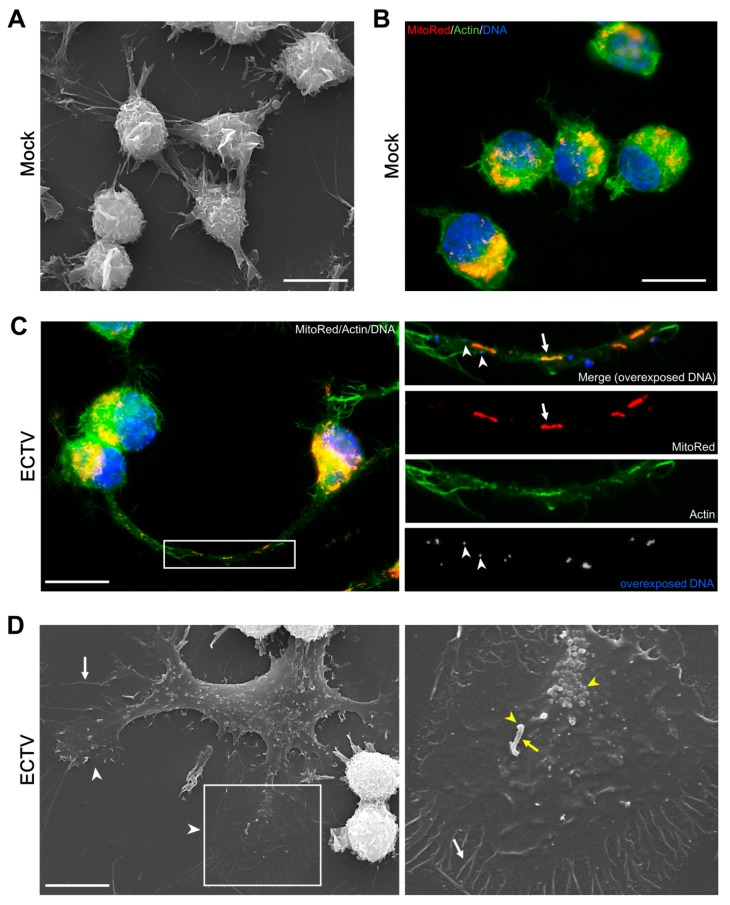
Morphology of RAW 264.7 cells during ECTV infection. Scanning electron micrograph of mock- (**A**) and ECTV-infected (**D**) cells; Actin cytoskeleton in mock- (**B**) and ECTV-infected (**C**) cells. White arrows indicate mitochondria in cytoplasmic corridor (**C**) or filopodia (**D**). White arrowheads indicate progeny virions (**C**) or lamellipodia (**D**). Yellow arrow indicates actin tail (**D**). Yellow arrowhead indicates progeny virions (**D**). Scale bar: 10 µm.

Visualization of morphological changes in ECTV-infected cells was performed using fluorescence staining of F-actin with phalloidin and subsequent fluorescent microscopy and SEM analysis. Infected RAW 264.7 macrophages (Figure 2A,B) strongly adhered to the slide surface (flattened cells), displaying a variety of morphological forms with actin-based cytoplasmic projections, including actin tails (short and thick), filopodia (short/long and thin), and long and wide extensions, referred as “cytoplasmic corridors” (Figure 2C,D) [25,26,27]. Furthermore, cytoplasmic projections contained ECTV particles [23,25] and mitochondria (Figure 2C).

### 3.3. Mitochondrial Network Morphology and Distribution in Murine Cell Lines during ECTV Infection

**Figure 3:**
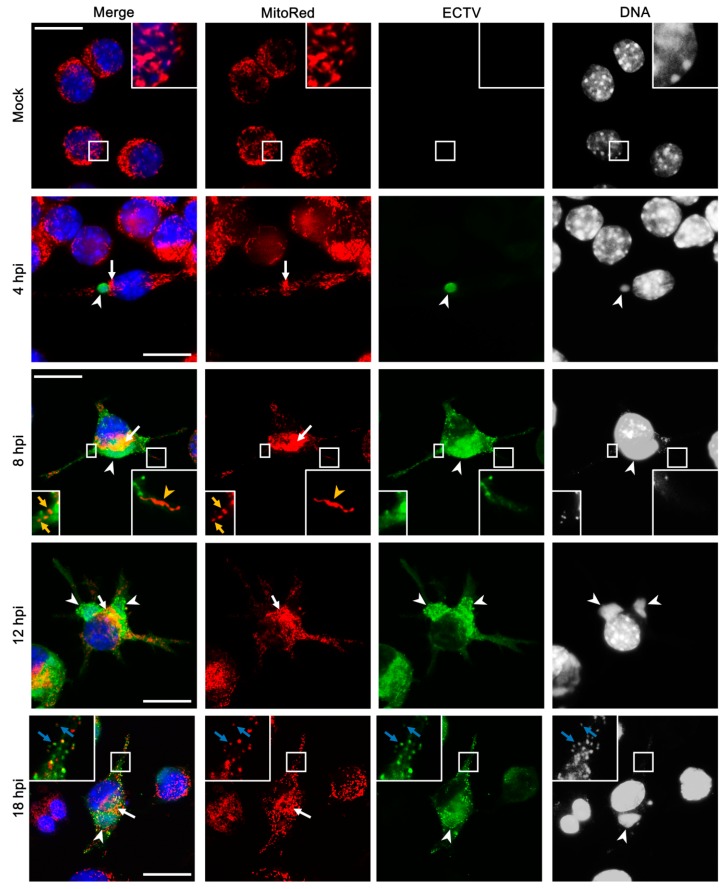
Mitochondrial network morphology in RAW 254.7 cells during ECTV infection. White arrows indicate mitochondria accumulated in close proximity to viral factories. White arrowheads indicate viral factories. Yellow arrows indicate fragmented mitochondria in cytoplasmic projection. Yellow arrowheads indicate elongated mitochondria in cytoplasmic projection. Blue arrows indicate fragmented, punctate mitochondria co-localized with progeny virions. Scale bar: 10 µm.

**Figure 4:**
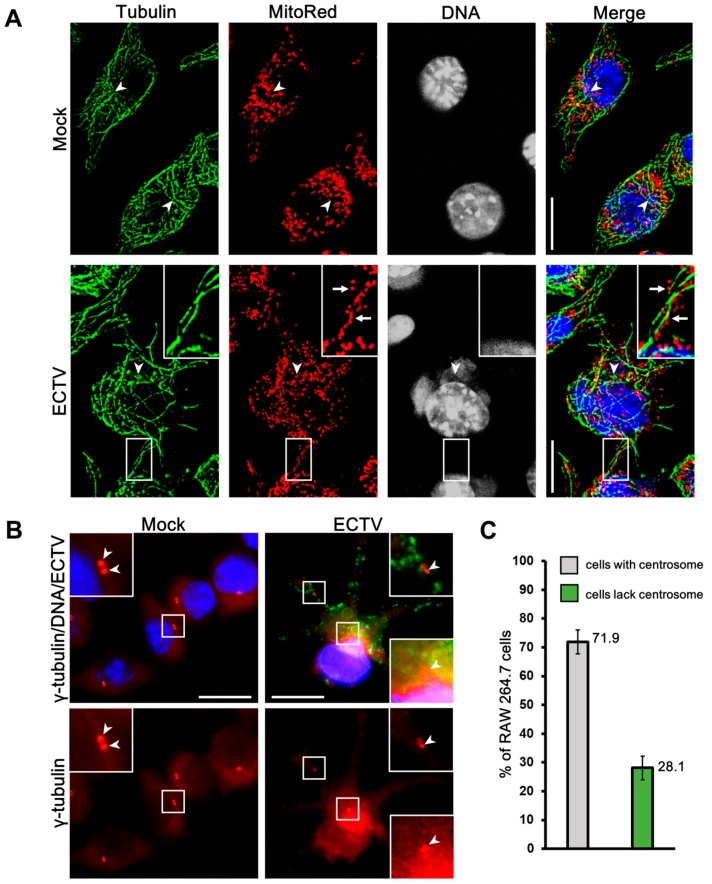
Tubulin cytoskeleton in RAW 264.7 cells at 18 hpi with ECTV. (**A**) α-tubulin staining. (**B**) γ-tubulin staining. (**C**) Percentage of virus-infected cells that show the centrosome or not. Arrowheads indicate microtubule organizing center (MTOC) with lack of mitochondria in mock cell or disappearance of MTOC that was replaced with viral factory and mitochondria in virus-infected cells (**A**) or centrioles (**B**). Arrows indicate punctate mitochondria on microtubules in mock-infected cells (**A**). Data from three independent experiments are presented as mean ± SD (*n* = 100 cells). Scale bar: 10 µm.

As described in our previous study [24], the branching structure of the mitochondrial network in uninfected L929 fibroblasts collapsed and became disorganized followed by destruction of mitochondrial tubules under the influence of ECTV at the late stages of infection (18 and 24 hpi). Mitochondria of uninfected RAW 264.7 cells were not branched, but had a regular distribution within the cytoplasm (Figure 3). The beginning of viral factory formation observed at 4 hpi was the earliest unequivocal evidence of the mitochondrial distribution changes within the cytoplasm of infected RAW 264.7 cells. At that time, small and round ECTV replication centers were observed in the perinuclear region with characteristic mitochondrial clustering in close proximity to these areas (Figure 3). A similar observation was made in L929 fibroblasts at 8 hpi with ECTV, when the viral factories occurred in most of the infected cells [24,25]. The manifestation of expanded (“bloated”) viral factories and the appearance of progeny virions within the cytoplasm of ECTV-infected RAW 264.7 and L929 cells at 8 and 12 hpi, respectively, began the progressive changes of mitochondrial network morphology during infection. Moreover, α-tubulin staining by immunofluorescence revealed the rearrangement of microtubules and disappearance of the microtubule organizing center (MTOC) during the late stages of ECTV infection in RAW 264.7 macrophages (Figure 4B vs. Figure 4A), as we previously described in L929 cells [24,25]. Further, the MTOC region in uninfected control cells was characterized by the absence of mitochondria in L929 [24] and RAW 264.7 cells (Figure 4A); however, the disappearance of this structure at later stages of ECTV infection led to the accumulation of mitochondria in this area [24].

Subsequent immunofluorescence staining using a marker of MTOC—γ-tubulin (which forms the centrosome and is responsible for microtubule nucleation)—confirmed the disappearance of the centrosome in ~30% of ECTV-infected RAW 264.7 cells (Figure 4B,C). The rest of the infected cells presented a weaker fluorescent signal from the centrosome or change of the centrosome location within the cytoplasm, in comparison to the control cells (Figure 4B). The centrosome containing a pair of centrioles was observed in the perinuclear region in uninfected control RAW 264.7 cells. Disappearance of the centrosome was previously described in L929 cells at the late stage of ECTV infection [25].

**Figure 5:**
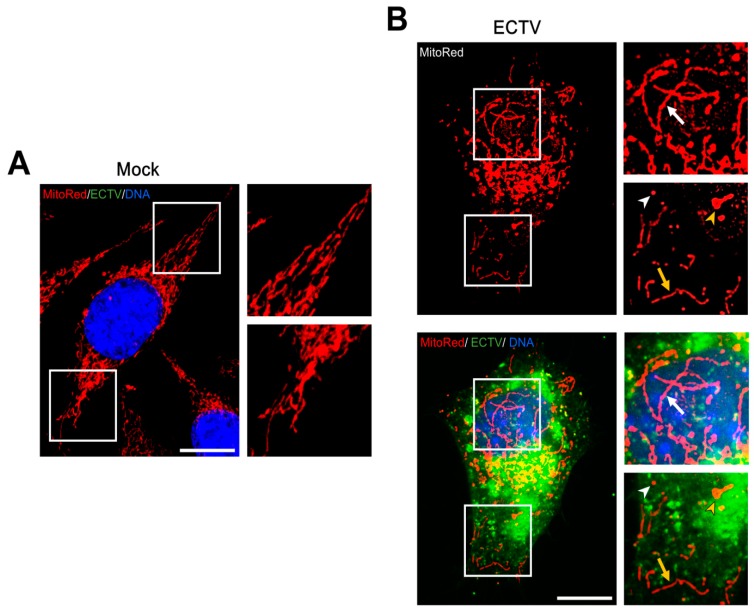
Mitochondrial network morphology in L929 cells at 12 hpi with ECTV. Interconnected mitochondrial network in mock-infected cells (**A**). Disorganized and fragmented mitochondrial network in ECTV-infected cells (**B**). White arrows indicate loose mitochondrial network (“loose net”). White arrowheads indicate small, punctate mitochondria. Yellow arrows indicate short, non-connected mitochondrial tubules. Yellow arrowheads indicate donut-like mitochondria. Scale bar: 10 µm.

**Figure 6:**
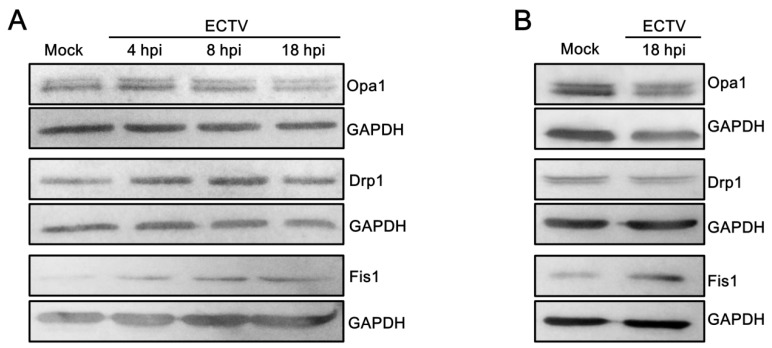

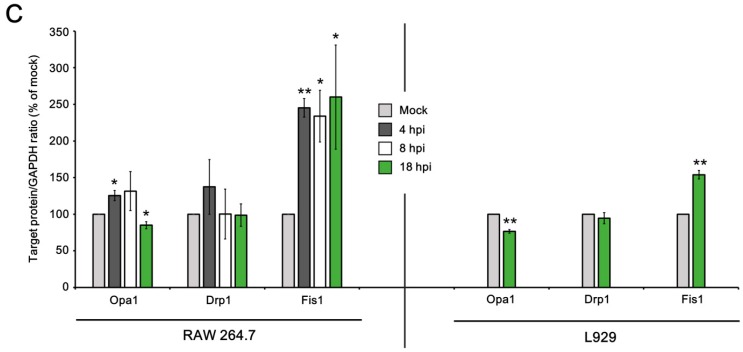
Level of mitochondrial fusion (optic atrophy protein 1, Opa1) and fission (dynamin-related protein 1, Drp1, and fission protein 1, Fis1) proteins in RAW 264.7 and L929 cells during ECTV infection. Representative Western blots of Opa1, Drp1, and Fis1 at 4, 8, 18 hpi in RAW 264.7 macrophages (**A**) and at 18 hpi in L929 fibroblasts (**B**). (**C**) Densitometry analysis of Opa1, Drp1, and Fis1 at 4, 8, 18 hpi in RAW 264.7 cells and at 18 hpi in L929 cells. The level of each protein was normalized to glyceraldehyde-3-phosphate dehydrogenase (GAPDH). Data from three independent experiments are presented as mean ± SD. Student’s *t*-test; * *p* ≤ 0.05; ** *p* ≤ 0.01.

Already from 12 hpi, the mitochondria of L929 cells were fragmented and disorganized with the loss of connection between themselves (Figure 5) [24]. The majority of mitochondria were tightly clustered in close association with viral factories (between virus replication centers and the nucleus or inside these “bloated” structures). However, some mitochondria were irregularly dispersed within the cytoplasm and displayed various forms, such as (a) a loose mitochondrial network (“loose net”), when the branched network was relaxed; (b) short, non-connected mitochondrial tubules; (c) donut-like mitochondria; and (d) single, small, circular mitochondria (punctate mitochondria) (Figure 5B), as was previously described at 18 hpi [24]. In contrast, mitochondrial disorganization and partial fragmentation in RAW 264.7 cells were observed starting from 8 hpi, but elongated mitochondrial tubules occurred as well (Figure 3). Interestingly, such mitochondrial elongation was not observed in uninfected control cells. Western blot analysis showed that the level of mitochondrial fusion protein Opa1 at 4 hpi with ECTV was slightly, but significantly higher by 26.4 ± 7.0% (*p* = 0.0227) in comparison to the control (Figure 6). The level of Opa1 at 18 hpi in RAW 264.7 cells was reduced by 15.1 ± 4.9% compared to the control. A similar decrease in Opa1 level by 23.4 ± 2.5% (*p* = 0.0039) was observed at 18 hpi in ECTV-infected L929 cells compared to in uninfected cells. Furthermore, the level of mitochondrial fission Fis1 protein increased 1.5- and 2.5-fold in L929 and RAW 264.7 cells, respectively, compared to the control (Figure 6).

**Figure 7:**
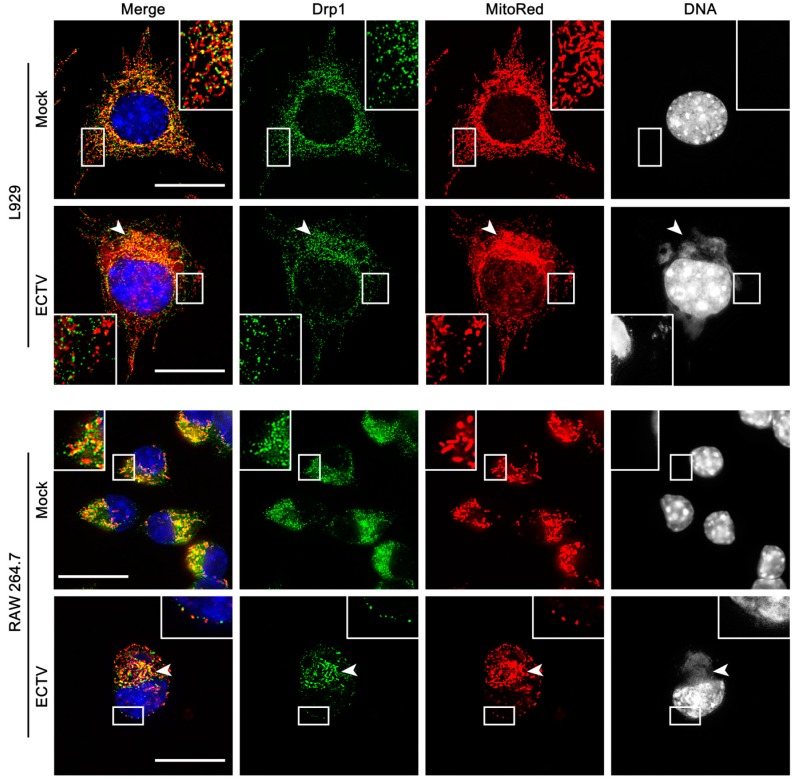
Distribution of Drp1 protein in L929 and RAW 264.7 cells at 18 hpi with ECTV. Mock-infected cells show regular distribution of Drp1. Arrowheads indicate accumulation of Drp1 in close association with viral factories that corresponds to the distribution of mitochondria. Scale bar: 10 µm.

Protein expression of Drp1 remained unchanged in both cell lines during ECTV infection compared to control, but its distribution within the cytoplasm was altered. Drp1 was distributed regularly within the cytoplasm in control cells, whereas in ECTV-infected L929 and RAW 264.7 cells, Drp1 localized in close association with viral factories, which corresponded to the mitochondrial accumulation around such compartments during infection (Figure 7).

### 3.4. Co-Localization of Punctate Mitochondria with Progeny ECTV Virions and Their Interaction with Cytoskeleton in Murine Cell Lines

**Figure 8:**
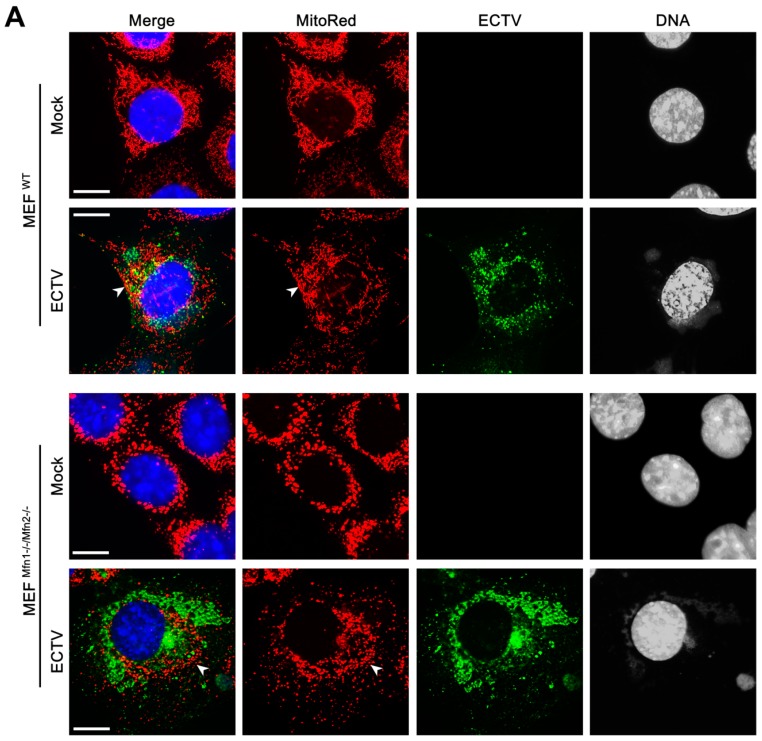

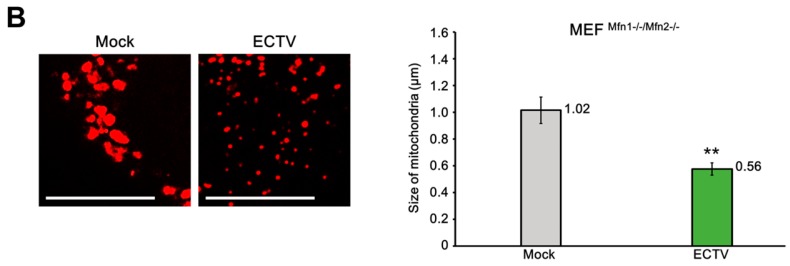
Hyperfragmentation of mitochondria in mouse embryonic fibroblast MEF^Mfn1−/−/Mfn2−/−^ cells at 18 hpi with ECTV. (**A**) Arrowheads indicate accumulation of mitochondria around viral factories in MEF^WT^ and MEF^Mfn1−/−/Mfn2−/−^ cells. (**B**) The mean size of mitochondria in mock- and ECTV-infected MEF^Mfn1−/−/Mfn2−/−^ cells. Data from three independent experiments are presented as mean ± SD (*n* = 100 mitochondria/cell, 50 cells were analyzed). Student’s *t*-test; ** *p* ≤ 0.01. Scale bar: 10 µm (**A**,**B**).

**Figure 9:**
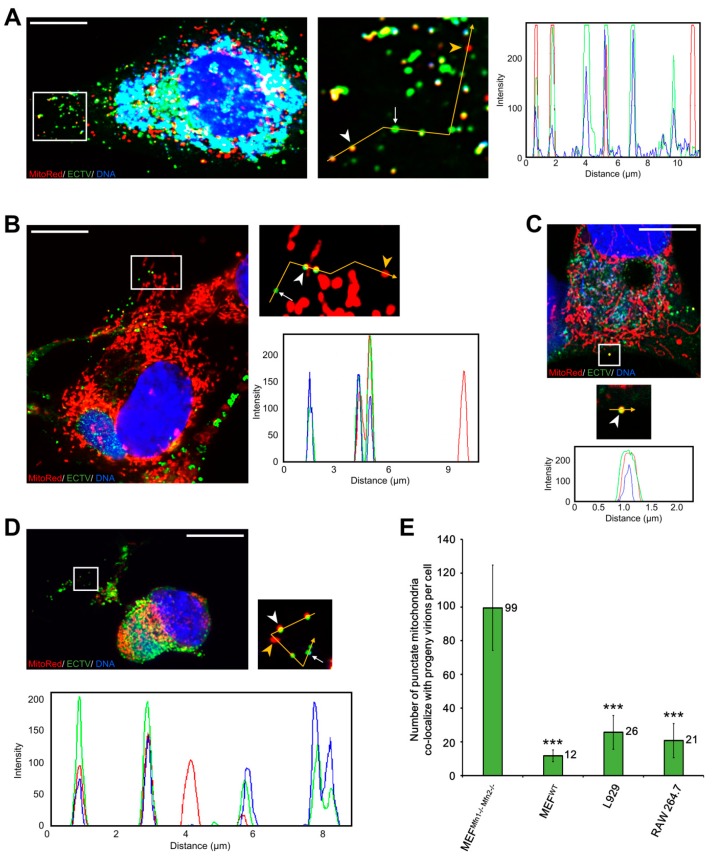
Co-localization of punctate mitochondria with ECTV progeny virions in MEF ^Mfn1−/−/Mfn2−/−^ (**A**), MEF^WT^ (**B**), L929 (**C**), and RAW 264.7 cells (**D**) at 18 hpi. The fluorescence intensity of mitochondria (red line), ECTV antigens (green line), and viral DNA (blue line) was measured along the yellow segmented arrows (**A**–**D**). White arrowheads indicate punctate mitochondria co-localized with virions; white arrows indicate virions alone; yellow arrowheads indicate mitochondria alone (**A**–**D**). (**E**) The mean number of punctate mitochondria co-localized with ECTV progeny virions in MEF ^Mfn1−/−/Mfn2−/−^, MEF^WT^, L929, and RAW 264.7 cells. Data from three independent experiments are presented as mean ± SD (*n* = 20 cells). Student’s *t*-test; *** *p* ≤ 0.001. Scale bar: 10 µm (**A**–**D**).

**Figure 10:**
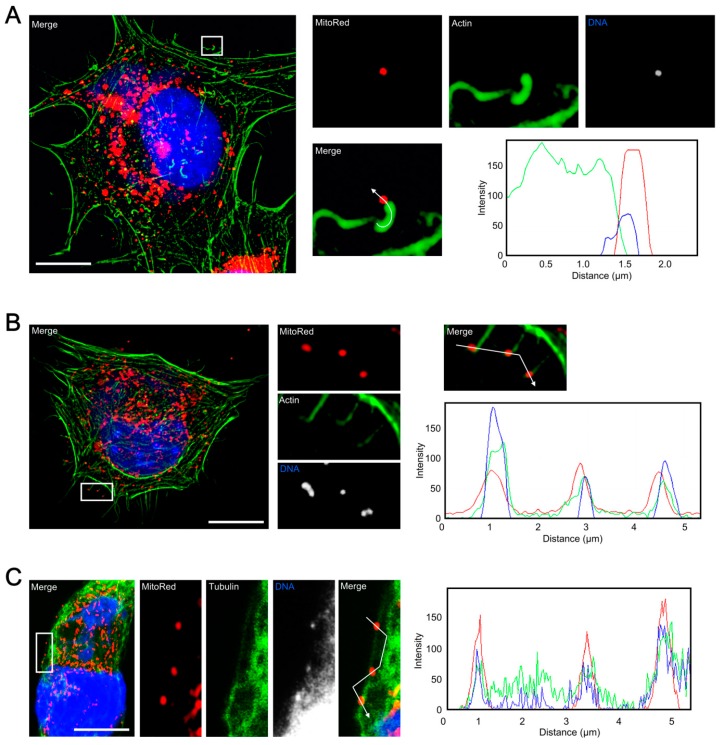
Punctate mitochondria co-localized with progeny virions interact with the cytoskeletal elements in ECTV-infected cells. (**A**) Mitochondrion co-localized with virion present on the top of the actin tail in MEF^Mfn1−/−/Mfn2−/−^ cells at 18 hpi. (**B**) Mitochondria co-localized with virions present on filopodia in L929 cells at 18 hpi. (**C**) Mitochondria co-localized with virions present on microtubules in L929 cells at 18 hpi. The fluorescence intensity of mitochondria (red line), actin (**A**,**B**) or tubulin (**C**) (green line), and viral DNA (blue line) was measured along the white segmented arrows. Scale bar: 10 µm (**A**–**C**).

To investigate the role of mitochondrial fusion during ECTV infection we used the MEFs^Mfn1−/−/Mfn2−/−^ cell line lacking both Mfn1 and Mfn2 and the MEFs^WT^ cell line as a control. The lack of Mfn1 and Mfn2 results in inhibition of the mitochondrial elongation process. Uninfected MEFs^WT^ exhibited an interconnected mitochondrial network typical for fibroblasts and similar to that observed in L929 cells, whereas mitochondria of MEFs^Mfn1−/−/Mfn2−/−^ were fragmented and clustered around the nucleus (Figure 8). At 18 hpi, the mitochondria of both cell types were disorganized and mostly accumulated near the viral factories (Figure 8A). ECTV-infected MEFs^Mfn1−/−/Mfn2−/−^ displayed hyperfragmentation of mitochondria (Figure 8B) with generation of numerous punctate organelles of which the majority co-localized with progeny ECTV virions (Figure 9A,E). Opposed to MEFs^Mfn1−/−/Mfn2−/−^, only a few punctate mitochondria co-localized with virions in ECTV-infected MEFs^WT^ (Figure 9B,E), L929 (Figure 9C,E), and RAW 264.7 cells (Figure 9D,E), as was also described in our previous study [24]. Such discrete mitochondrial interaction with virions was observed on the top of ECTV-induced actin tails and filopodia, as well as on microtubules (Figure 10).

**Figure 11:**
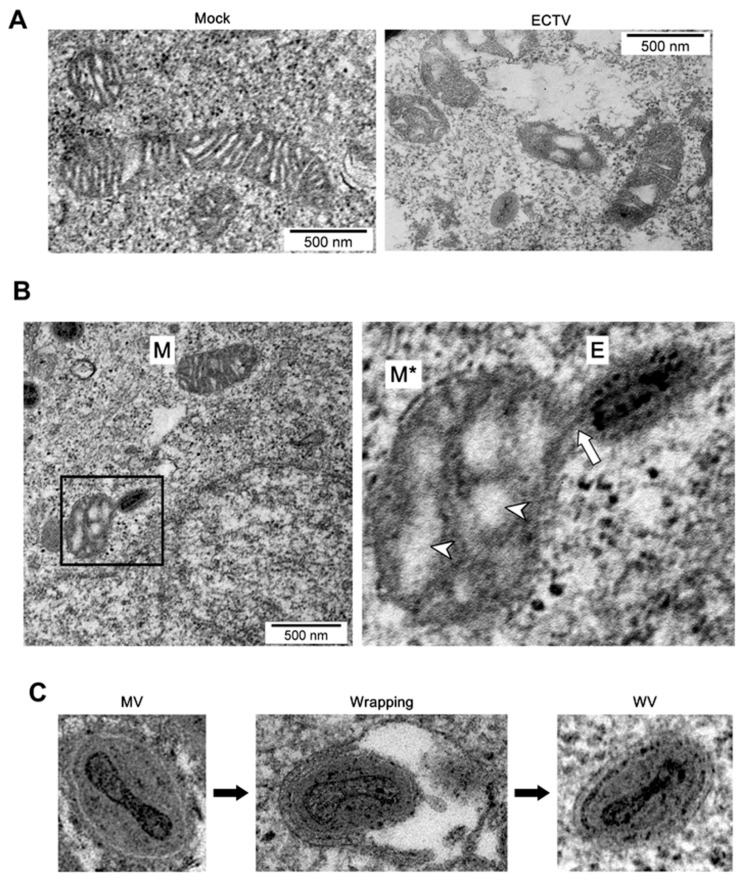
ECTV-infection alters mitochondrial structure in permissive cells. (**A**) Transmission electron micrograph showing mitochondria in control and ECTV-infected L929 cells. (**B**) Interaction between mitochondrion and virion via membrane bridge in L929 cells at 18 hpi. Arrow indicates membrane bridge. Arrowheads indicate swollen mitochondrial matrix. (**C**) Types of intracellular ECTV virions observed in RAW 264.7 cells at 18 hpi. E—ECTV virion; M—mitochondrion; M*—swollen mitochondrion; MV—mature virion; WV—wrapped virion. Scale bar: 500 nm.

To further scrutinize the mitochondria–ECTV interaction we used transmission electron microscopy (TEM). Figure 11A shows a mitochondrion (~470 × 300 nm) in L929 cells structurally connected to ECTV virion (~250 × 125 nm) via some form of membrane bridge. The structure of such mitochondria was altered with a clearly swollen matrix (Figure 11A). Viral particles connected to mitochondria were characterized by the presence of only one membrane, typical for mature virions (MVs) of poxviruses (Figure 11B).

### 3.5. Mitochondrial Physiology in Murine Cells during ECTV Infection

Because the mitochondrial networks in RAW 264.7 and L929 cells were disorganized and partially fragmented at later stages of infection, we hypothesized that infection with ECTV can negatively impact the mitochondrial physiology. Taking into account the divergences in the replication kinetics of ECTV between fibroblasts and macrophages, appropriate time points after infection of each cell line were selected for mitochondrial physiology analysis.

**Figure 12:**
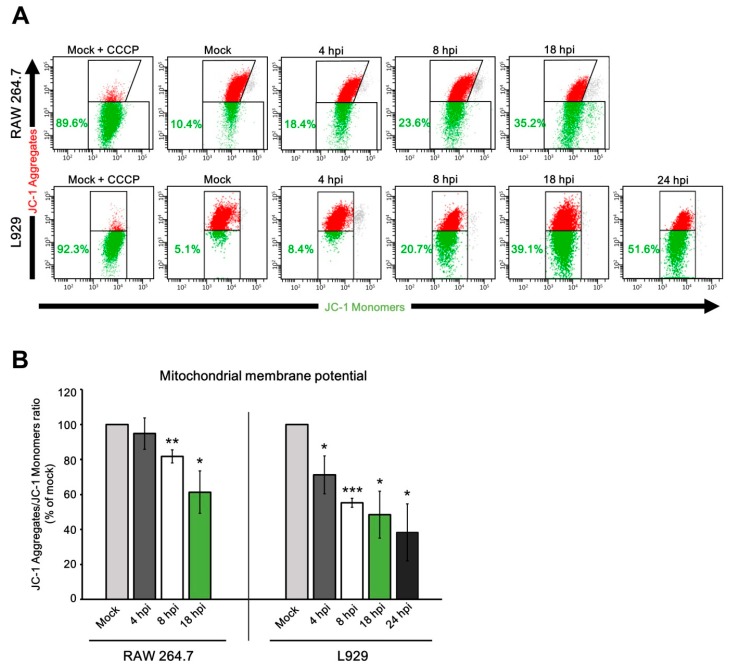
Mitochondrial membrane potential (MMP) in RAW 264.7 and L929 cells during ECTV infection. (**A**) Representative double fluorescence dot plots of flow cytometry analysis of 5,5′,6,6′-tetrachloro-1,1′,3,3′-tetraethylbenzimidazol-carbocyanine iodide (JC-1) staining in RAW 264.7 and L929 cells during ECTV infection. Red populations indicate cells with high MMP. Green populations indicate cells with depolarized MMP. (**B**) Mean JC-1 Aggregates/JC-1 Monomers ratio at 4, 8, and 18 hpi in RAW 264.7 macrophages and at 4, 8, 18, and 24 hpi in L929 fibroblasts. Data from three independent experiments are presented as mean ± SD (*n* = 10,000 cells). Student’s *t*-test; * *p* ≤ 0.05; ** *p* ≤ 0.01; *** *p* ≤ 0.001.

Due to the critical role of MMP in mitochondrial homeostasis, we evaluated MMP in uninfected and infected L929 and RAW 264.7 cells using JC-1 staining and subsequent flow cytometry analysis (Figure 12A). At 4 hpi with ECTV, MMP was decreased by almost one third (*p* ≤ 0.05) in L929 fibroblasts, compared to the uninfected control; however, no statistically significant difference in MMP was reported in infected RAW 264.7 macrophages at 4 hpi (Figure 12B). The first statistically significant reduction of MMP in RAW 264.7 cells occurred at 8 hpi (by 18.3 ± 3.7%; *p* = 0.013), whereas in L929 cells, MMP declined by 45% (*p* ≤ 0.001) compared to control. At 18 hpi, MMP in RAW 264.7 cells was lower by 40% (*p* ≤ 0.01) compared to control, while in L929 cells MMP was reduced by more than 50% (*p* ≤ 0.05). At 24 hpi, MMP in L929 fibroblasts was decreased by 60%. Generally, a considerable percentage of cells with depolarized MMP was observed in both cell lines and the results showed increasing numbers of cells with low MMP as the infection progressed (Figure 12B).

**Figure 13:**
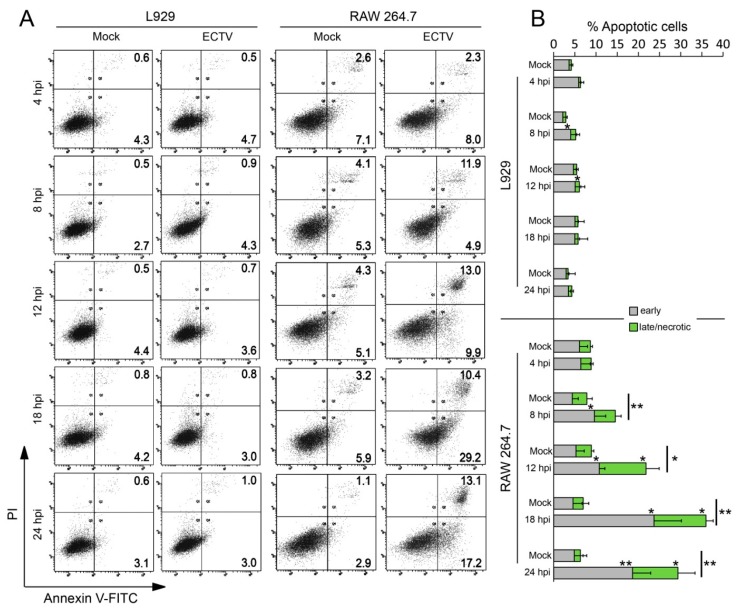
Apoptotic rate in RAW 264.7 and L929 cells during ECTV infection. (**A**) Representative double fluorescence dot plots of flow cytometry analysis of Anexin V- fluorescein isothiocyanate (FITC) and propidium iodide (PI) staining in RAW 264.7 and L929 cells at 4, 8, 12, 18, 24 hpi with ECTV. (**B**) The percentage of early and late apoptotic or necrotic cells. Student’s *t*-test; * *p* < 0.05, ** *p* < 0.01.

In order to verify whether the collapse of MMP in ECTV-infected cells is associated with cell death, we measured the apoptotic rate of L929 and RAW 264.7 cells. Our result showed that ECTV infection does not induce cell death in L929 fibroblasts; however, the apoptotic/necrotic rate in RAW 264.7 macrophages reached ~40% at the late stages of infection (Figure 13).

**Figure 14:**
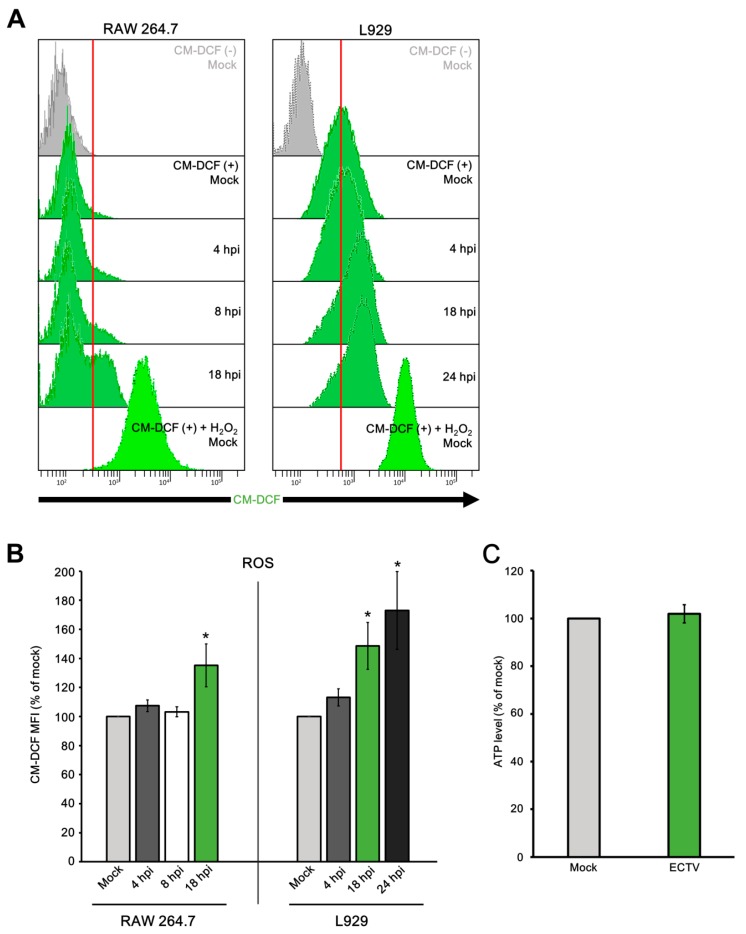
Level of reactive oxygen species (ROS) and ATP in RAW 264.7 and/or L929 cells during ECTV infection. (**A**) Representative histograms of flow cytometry analysis of 5-(6)-chloromethyl-2′,7′-dichlorofluorescein (CM-DCF) staining in RAW 264.7 and L929 cells during ECTV infection. Grey populations indicate no-stain mock cells. Mock cells stained with CM-DCF were used as a negative control. Mock cells treated with H_2_O_2_ and stained with CM-DCF were used as a positive control. (**B**) Mean fluorescence intensity (MFI) of CM-DCF at 4, 8, and 18 hpi in RAW 264.7 macrophages and at 4, 18, and 24 hpi in L929 fibroblasts. Data from three independent experiments are presented as mean ± SD (*n* = 10,000 cells). Student’s *t*-test; * *p* ≤ 0.05. (**C**) Level of ATP in control and ECTV-infected L929 fibroblasts at 18 hpi.

Reduction in MMP is accompanied by ROS generation; therefore, in the next step we performed measurement of the ROS level in L929 and RAW 264.7 cells using CM-H_2_DCFDA labeling and flow cytometric analysis (Figure 14A). At 4 hpi, there were no statistically significant changes in ROS production between infected and control cells in both cell lines (Figure 14B). However, fibroblasts and macrophages presented an increased level of ROS at the later stages of infection, compared to the uninfected control (*p* ≤ 0.05). The production of ROS was higher by approximately 35% in RAW 264.7 at 18 hpi, and by ~50% and ~70% in L929 cells at 18 and 24 hpi, respectively (Figure 14B).

Reduction in MMP results in decreased energy provision, so we next examined how ECTV infection influences ATP production in permissive cells. Interestingly, in ECTV-infected L929 fibroblasts at 18 hpi, the level of ATP remained unchanged compared to that in uninfected control cells (Figure 14C).

**Figure 15:**
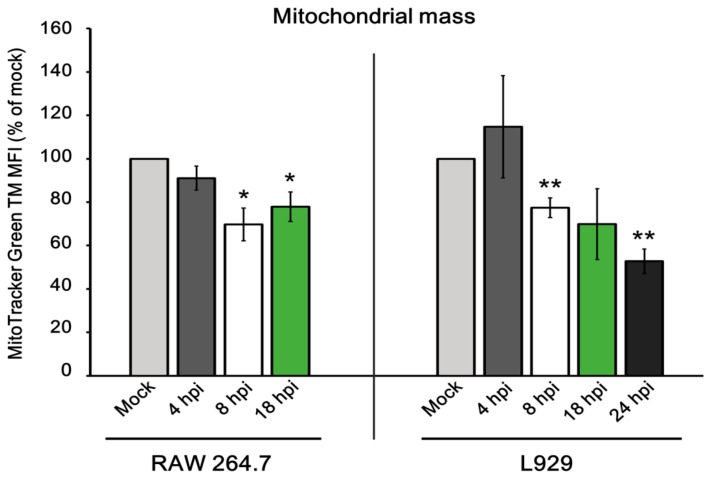
Mitochondrial mass in RAW 264.7 and L929 cells during ECTV infection. Mean fluorescence intensity (MFI) of MitoTracker Green TM at 4, 8, and 18 hpi in RAW 264.7 macrophages and at 4, 8, 18, and 24 hpi in L929 fibroblasts. Data from three independent experiments are presented as mean ± SD (*n* = 10,000 cells). Student’s *t*-test; * *p* ≤ 0.05; ** *p* ≤ 0.01.

**Figure 16:**
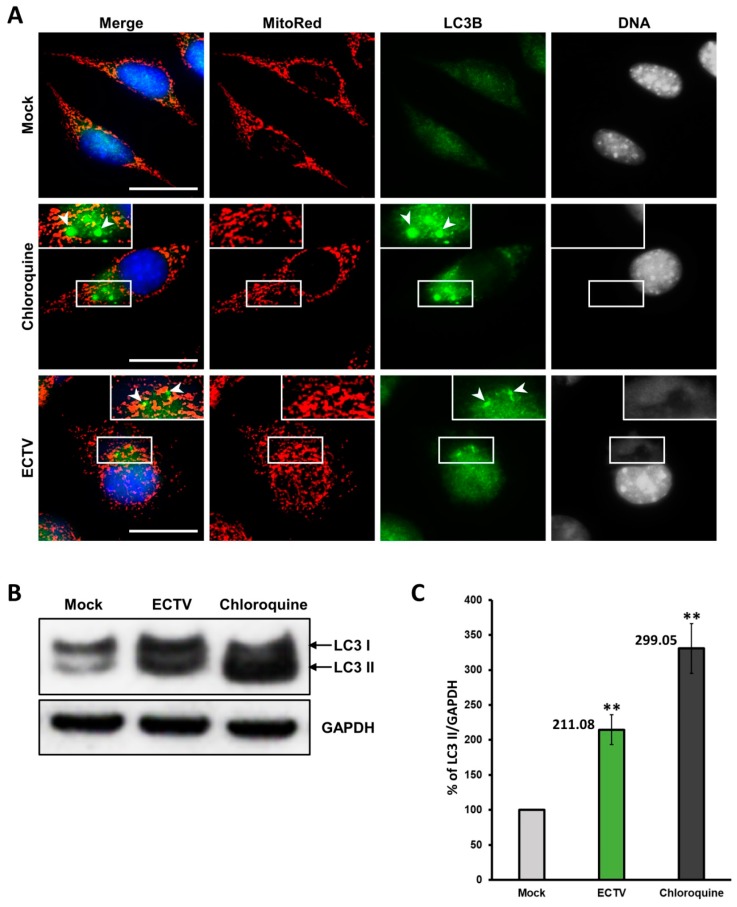
ECTV induces autophagy in later stages of infection in L929 fibroblasts. Uninfected cells pretreated with 30 µM chloroquine for 16 h were used as a positive control. (**A**) The microtubule-associated light chain 3B (LC3B) staining; (**B**) representative Western blots of LC3B at 18 hpi; (**C**) densitometry analysis of LC3 II at 18 hpi. The level of protein was normalized to GAPDH. Data from three independent experiments are presented as mean ± SD. Student’s *t*-test; ** *p* ≤ 0.01. Scale bar: 20 µm (**A**).

Since ECTV-infected cells exhibited an imbalance in fusion–fission processes, we finally addressed mitochondrial mass in such cells. The mitochondrial mass measurement was performed using flow cytometric analysis of live L929 and RAW 264.7 cells labeled with MitoTracker Green FM. At 4 hpi with ECTV, there were no statistically significant changes in mitochondrial mass in both cell lines compared to the control (*p* ≤ 0.05). From 8 hpi, L929 and RAW 264.7 cells were characterized by decreased mitochondrial mass until the later stages of infection (Figure 15). Reductions by ~30% at 8 hpi and ~20% at 18 hpi were noticed in RAW 264.7 cells, whereas decreases by ~20% at 8 hpi, ~30% at 18 hpi, and ~50% at 24 hpi were observed in L929 cells. Due to the loss of mitochondrial mass in ECTV-infected cells, we additionally verified the presence of autophagy during infection. Fluorescence microscopy analysis showed intensified aggregates of LC3B in infected cells (Figure 16A). Additionally, Western blot analysis indicated a significantly (*p* ≤ 0.01) higher level of LC3B II (active form of LC3B) in virus-infected L929 fibroblasts at 18 hpi, compared to the control (Figure 16B,C). Both results strongly suggest the induction of autophagy.

## 4. Discussion

Structural and functional changes in the cell caused by viral infection are referred to as cytopathic effect (CPE). The nature of CPE in a cell culture infected with poxvirus depends on the type of virus, type of cells or tissue, and the MOI [28,29]. Therefore, in order to link CPE, including alterations in mitochondrial network morphology and physiology, to the particular stages of infection, we first evaluated the kinetics of ECTV replication in RAW 264.7 and L929 cells. Despite using the same MOI of 5, ECTV replicated faster in RAW 264.7 macrophages than in L929 fibroblasts, as evidenced by replication center formation. Our data confirmed that the duration of the poxvirus replication cycle is cell type dependent [30]. The differences between RAW 264.7 and L929 cells in the kinetics of viral replication may arise from the higher tropism of ECTV to macrophages or greater efficiency of virus entry into this cell type. Studies have shown that mature virions (MV) of VACV use a distinct form of macropinocytosis for host cell entry [31]. Moreover, MV correspond in size to apoptotic bodies, and, as such, may also be engulfed through macropinocytosis by specialized cells such as macrophages [31,32,33]. Others have hypothesized that VACV virions enter host cells by mimicking apoptotic bodies, leading not only to effective macropinocytosis, but also to the avoidance of immune recognition [31]. This is due to the fact that macropinocytosis of apoptotic debris suppresses the activation of innate immune responses [34]. It can be assumed that RAW 264.7, as phagocytic cells, may exhibit a greater ability to macropinocytose viral particles, unlike non-specialized L929 fibroblasts.

Alterations in cell shape and size are strictly related to rearrangement of the cytoskeleton, which is responsible for intracellular distribution of organelles, including mitochondria. Mitochondrial organization within the cytoplasm contributes to their function and is essential for cell survival [35]. Our results indicate that ECTV infection causes cytoskeleton rearrangement in RAW 264.7 macrophages, which is expressed by changes in cell shape and occurrence of cytoplasmic projections. Similar results were shown in our previous study on ECTV-infected L929 [25] and BALB 3T3, HeLa, and Vero cells [23]. Other studies also have shown similar observations during VACV, VARV, monkeypox virus (MPXV) [36], and myxoma virus (MYXV) [27] infection. The observed presence of ECTV particles on or in cytoplasmic extensions of RAW 264.7 in the current study suggests the role of these structures in cell-to-cell viral spreading that has been confirmed by numerous studies on different types of viruses belonging to distinct families, such as *Poxviridae* [23,25,27,37,38], *Reoviridae* [39], and *Togaviridae* [40]. We can speculate that the formation of “cytoplasmic corridors” by ECTV-infected cells allows for direct transportation of viral particles between infected and uninfected cells, avoiding contact of cell membranes. Thus, virus entry into host cells could be simplified, permitting earlier initiation of virus replication. Furthermore, the lack of contact of viral particles with the extracellular environment may provide protection against unfavorable conditions outside the cell, including the antiviral immune mechanisms in vivo.

Proper mitochondrial network morphology and distribution within the cytoplasm is essential for cell survival and contributes to organelle function. Immunofluorescence and Western blot analysis revealed that mitochondrial fusion–fission processes were altered in ECTV-infected RAW 264.7 and L929 cells, compared to in uninfected control cells. The mitochondrial network of these cells was partly fragmented and disorganized. Our present and previous studies indicate that formation of viral factories relates to the beginning of changes in localization of mitochondria within the cytoplasm in RAW 264.7 and L929 cells [24]. Mitochondria of infected cells were clustered in close proximity to the early ECTV replication centers. Our data show that the MTOC—a structure from which microtubules emerge—partly or completely disappears in ECTV-infected RAW 264.7 macrophages, as was previously described in L929 fibroblasts [24,25] and during VACV infection of Hela cells [41]. Viral factories form in the vicinity of the MTOC, suggesting the role of microtubules in the transport of poxvirus particles [24,25,41]. It is likely that localization of mitochondria near viroplasms (viral factories) in ECTV-infected cells may compensate for the loss of the MTOC.

Rojo et al. [42] have shown that mitochondria in Vero cells also aggregated near viral factories during infection with African swine fever virus (ASFV)—another large DNA virus predominantly replicating within the cytoplasm. Rojo et al. [42] suggested the role of mitochondria in supplying energy for the ASFV morphogenetic processes [42]. Other studies confirmed this fact by showing that ATP is required for the wrapping of ASFV progeny particles [43,44]. Taken together, the accumulation of mitochondria in close proximity to the ECTV replication centers suggests that mitochondria are active and probably supply ATP for replication of viral DNA and morphogenesis of progeny virions. According to Chang et al. [45] VACV-infected cells produced two times more ATP than control cells, and a high level of ATP is necessary to drive efficient virus replication. Moreover, an elevated oxygen consumption rate (ATP synthesis) during VACV infection was also confirmed in later studies [46]. Surprisingly, the level of ATP in ECTV-infected L929 cells did not decrease, suggesting that despite alteration in mitochondrial physiology, these organelles were still able to produce ATP, therefore delaying induction of apoptosis.

Recent studies have indicated that infection with DNA and RNA viruses such as hepatitis B virus (HBV) [47], human cytomegalovirus (HCMV) [48], Epstein–Barr virus (EBV) [49], hepatitis C virus (HCV) [50], influenza A virus (IAV) [51], pseudorabies virus (PRV) [52], and Dengue virus (DENV) [53] is able to affect the structure of the mitochondrial network, leading to its fragmentation. Kim et al. [47] found that HBV and its encoded HBx protein promoted perinuclear clustering of mitochondria and triggered mitochondrial translocation of Drp1, resulting in mitochondrial fission. Although the present study shows that the level of Drp1 protein was unchanged during ECTV infection, the localization of this protein was altered. Drp1 accumulated near mitochondria and viral factories, suggesting that a similar mechanism may exist in ECTV-infected cells. To the best of our knowledge, there is no report describing a poxviral protein responsible for mitochondrial dynamics alteration. However, poxviruses encode mitochondria-localized proteins, including EVM025 from ECTV [19], F1L from VACV [54], and M11L from MYXV [55] that localize to the mitochondria through the C-terminal transmembrane domain, where they can carry out their anti-apoptotic functions. Furthermore, mutation of some ECTV proteins appears to have a different role in in vivo and in vitro conditions. For example, A36^YdF^ ECTV, which has mutations at the two critical tyrosine residues Y112 and Y132 in the A36 protein, exhibits disrupted actin-based motility and shows impaired release in vitro, but relatively normal dissemination in vivo [56].

Apart from partial fragmentation of the mitochondrial network, the ECTV-infected cells displayed elongated mitochondria as well. A study by Castanier et al. [57] has shown that excessive mitochondrial fusion (elongated, branched mitochondria) increases the interaction of MAVS with other molecules involved in the RLR-dependent signaling pathway, which leads to production of anti-viral factors—type I IFNs and pro-inflammatory cytokines upon poly(I:C) stimulation or Sendai virus infection. Conversely, fragmented mitochondria cause a decrease in ability to transmit the signals for type I IFN production. Inhibition of mitochondrial fragmentation by silencing Drp1 during HCV infection enhanced type I IFN production, suggesting that HCV-induced mitochondrial fission contributes in part to the evasion of the innate immune system [50]. Based on our results, we speculate that excessive mitochondrial fission observed during the late stages of ECTV infection would serve to perturb the cellular antiviral defense. Because there is no data describing an ECTV-encoded protein that stimulates mitochondrial fragmentation, it is likely that mitochondrial fission is related to the progressive damage of mitochondria during infection, as a “side effect” of the virus activity. Nevertheless, even accidental activation of mitochondrial fission would presumably lead to a blockade of MAVS downstream signaling, resulting in reduced IFN synthesis and sustained viral infection. Recently, [58] it has been shown that ECTV infection of GM-CSF-derived bone marrow cells (composed of conventional dendritic cells and macrophages) induces down-regulation of genes involved in the RIG-I-MAVS signaling pathway.

The use of the cytoskeleton for movement of viral particles was previously described during infections with orthopoxviruses, such as ECTV [23], VACV [59], VARV, and MPXV [36], as well as other distinct poxviruses, e.g., Yaba-like disease virus (YLDV) [60], swinepox virus (SWPV) [61], and MYXV [27]. Intracellular transport of virions along microtubules and actin polymerization to form virus-induced actin comet tails are necessary for viral spreading and require the energy from ATP hydrolysis. The major cellular source of ATP is mitochondria; this would suggest that punctate mitochondria supply energy for the transportation of progeny virions inside and outside the cell through the cytoskeleton. Mitochondrial fragmentation facilitates transportation of mitochondria along microtubules, whereas motility of strongly elongated organelles is prevented [62,63]. We can speculate that the selective interaction of progeny ECTV virions with only punctate mitochondria in the infected cells provides efficient transport of viral particles along the cytoskeletal elements. Considerably lower numbers (statistically significant; *p* ≤ 0.05) of this type of mitochondria interacted with ECTV in L929 (25.1 ± 10.0), RAW 264.7 (20.8 ± 10.1) and MEFs^WT^ (11.8 ± 3.5) compared to in MEFs^Mfn1−/−/Mfn2−/−^ (99.5 ± 25.2) which are defective in the mitochondrial fusion process. This suggests that mitochondrial elongation prevents excessive mitochondrial fission and may constitute a protective mechanism against viral transport and spreading. However, this assumption requires further investigation. The presence of punctate mitochondria co-localized with ECTV virions on the top of actin tails suggests that the intact mitochondria are released outside the cell, a rather controversial issue. However, Maeda and Fadeel [64] have recently shown that mitochondria are released from necroptotic cells before the disruption of the plasma membrane, indicating that this is an active process. Mitochondria released in such a manner maintain their function with no mtDNA removal and can affect the cytokine production by macrophages and cell maturation of antigen-presenting dendritic cells [64].

TEM analysis showed that mitochondria in L929 cells formed a structural connection with ECTV virions via a membrane bridge. It is possible that the membrane bridge structure may have an origin in the mitochondrial outer membrane, inner membrane, or both. In any case, the pivotal issue here concerns definition of the role of this structure. Interestingly, the mitochondrion-connected viral particle had only one membrane, a characteristic typically associated with mature virions (MV) of poxviruses. Some MVs are wrapped in additional, cell-derived membranes to become triple-membrane particles called wrapped virions (WVs) [65]. As it currently stands, the modified trans-Golgi or endosomal membrane is the origin of the viral envelope [65,66]. Due to the fact that mitochondria contain two membranes—inner and outer—it is not excluded that ECTV virions could utilize mitochondrial membrane/s for viral envelope formation. Hawes et al. [67] have shown that the thickness of the African swine fever virus (ASFV) envelope is very similar to that of a single lipid bilayer of mitochondria and other cellular organelles. According to our study, there is no reason to reject the hypothesis about mitochondrial origin of the ECTV envelope.

The evaluation of mitochondrial physiology indicates that ECTV infection contributes to an ongoing reduction of MMP in L929 and RAW 264.7 cells. Our results of mitochondrial fission in ECTV-infected cells confirm that the loss of mitochondrial membrane potential is related with mitochondrial dysfunction and fragmentation [68,69]. Further, we show that alterations in MMP appeared earlier and were higher in fibroblasts than in macrophages. This shows that RAW 264.7 cells are more resistant to the loss of MMP triggered by ECTV infection. Previous studies of mechanisms by which poxviruses regulate apoptosis have indicated that ECTV and VACV infection do not affect mitochondrial membrane potential [19,20,21]. This stands in contrast to our data and can be related to different fluorescent probes applied to measure MMP in our study. Additionally, the dissimilarity between our results and others in the level of MMP may arise from differences in the time post-infection when the measurements were taken. No changes in MMP were noted at 6–8 hpi with ECTV or VACV; however, further observations were not conducted [19,20,21]. The only exceptions are results reported from experiments performed by Melo-Silva et al. [70] in which MMP declined at 24 hpi with ECTV compared to control, and no differences were observed at 8 hpi. Therefore, it can be assumed that similar alterations could have been observed during the other cited studies.

The collapse of MMP is linked to mitochondrial damage and is often a manifestation of apoptosis activation. In this context, occurrence of apoptosis in ECTV-infected L929 and RAW 264.7 cells was determined. Our results indicated no apoptotic changes in L929 fibroblasts; however, RAW 264.7 macrophages exhibited an apoptotic rate of ~40%. The discrepancy between results may arise from the different properties of cell lines that influenced the duration of the virus replication cycle. Higher tropism of ECTV to macrophages or greater efficiency of virus entry into this cell type could be a reason for both faster virus replication and apoptosis activation. On the other hand, lack of cell death in L929 cell during ECTV infection suggest no virus-associated cytotoxicity effect on this cell line at MOI = 5, even in the late stages of infection.

Increased levels of ROS were evident in both L929 and RAW 264.7 cells during the later stages of infection with ECTV. Mitochondria are the main source of ROS both in normal and pathological conditions but are simultaneously one of the most endangered ROS targets [71]. Zorov et al. have shown that higher levels of ROS result in the loss of MMP, which consequently leads to mitochondrial permeability transition and damage [72]. In turn, defective mitochondria generate even more ROS which contributes to even greater damage to mitochondria. Similar results were obtained during HCV infection [73]. Intended inhibition of ROS production in HCV-infected cells resulted in both a reduction of ROS level and prevention of mitochondrial degradation. Based on these reports, it can be suggested that the increased level of ROS generated during ECTV infection triggers mitochondrial damage, manifested by the loss of MMP and mitochondrial fragmentation.

The molecular mechanism of ECTV-induced attenuation of MMP and progression of mitochondrial fragmentation is not known and should be elucidated in future studies to extend our knowledge about the implications of mitochondrial dynamics in orthopoxviral pathogenesis. We speculate that ECTV may encode protein(s) that accumulate in mitochondria and therefore affect mitochondrial functions. It has been shown that the PB1-F2 protein of the influenza A virus constitutively localizes to the mitochondrial inner membrane space via Tom40 channels, and its accumulation leads to remarkable mitochondrial fragmentation due to reduced MMP [74]. MMP reduction was correlated with impaired mitochondrial-mediated innate immune responses, including the RIG-I signaling pathway and NLRP3 inflammasomes [74].

Our results show that later stages of ECTV infection are critical to the reduction of mitochondrial mass in L929 and RAW 264.7 cells. It appears that there is successive mitochondrial damage, presumably as a result of disruption of mitochondrial function during infection. Defective mitochondria can be targeted for degradation via selective (mitophagy) or nonselective autophagy, due to the activation of mitochondrial permeability transition and/or loss of mitochondrial membrane potential [75,76,77]. Our current and previous studies have shown the presence of autophagy in ECTV-infected L929 cells [77], which suggests that some type of autophagy may be responsible for mitochondrial removal in infected murine cell lines, manifested by the decrease of mitochondrial mass.

Mitophagy is tightly related to mitochondrial dynamics, and together they are identified as the key pathways in mitochondrial quality control [78]. A number of studies have shown that mitochondrial fission is responsible for segregation and elimination of damaged organelles out of the cell, pointing to the protective role of this process [79,80]. Fragmented mitochondria with irreparable defects constitute a risk to cell survival, due to the harmful effects of ROS production [80]. Oxidative stress generates damage to DNA, proteins, and lipids, consequently leading to cell death. During poxviral infection, including ECTV, prolonged maintenance of the vital functions by the host cell is critical for efficient viral replication. Poxviruses encode anti-apoptotic proteins [19,21]; therefore, induction of cell death is not supportive of viral survival. Kim et al. [47,50] have demonstrated that HBV and HCV infection lead to autophagy (mitophagy) activation that is presumably a counteraction against virus-induced apoptosis. HBV or HCV infection stimulates synthesis of Drp1 and triggers its translocation to mitochondria, leading to mitochondrial fission and then mitophagy. Intended inhibition of mitochondrial fragmentation by silencing of Drp1 resulted in reduction of mitophagy and enhancement of apoptosis [47,50]. In the light of findings such as fragmentation of mitochondrial network, mitochondrial mass reduction, and presence of autophagy, it is likely that ECTV infection contributes to the active process of mitochondria removal from L929 and RAW 264.7 cells leading to inhibition of apoptosis. We cannot rule out the possibility that mitochondrial elimination impairs MAVS-dependent anti-viral immunity and promotes virus infection as well [81]. Higher ROS levels increase MAVS downstream signaling resulting in elevated type I IFN synthesis and limited development of virus infection [81]. Based on our results, removal of mitochondria from ECTV-infected cells may not only protect from host cell death, but may also allow the virus to avoid mitochondrial-dependent immunity. It should be noted that the increased level of ROS during the late stages of ECTV infection would theoretically indicate an opposite effect; however, the inability to eliminate damaged mitochondria could possibly lead to even greater amount of ROS than was observed.

## 5. Conclusions

Our study shows that ECTV infection alters the mitochondrial network morphology and distribution, especially through fragmentation of mitochondria and their accumulation in close proximity to the viral factories. Mitochondrial fission at the later stages of infection led to the formation of small, punctate mitochondria that co-localized with progeny virions and were found on cytoskeleton components, like actin tails, filopodia, and microtubules. Mitochondrial function in infected cells was altered, and involved the decrease of mitochondrial membrane potential, increase of ROS level, reduction of mitochondrial mass, and presence of autophagy, suggesting degradation of mitochondria and their elimination from the cell at the later stages of infection. ECTV replicated at a higher rate in RAW 264.7 cells and consequently induced more rapid changes in mitochondrial network morphology and distribution than in L929 cells. Disruption of mitochondrial function occurred at a later time point in macrophages compared to in fibroblasts. The observed alterations in the mitochondrial network morphology and organization as well as mitochondrial function during ECTV infection provide suitable conditions for viral replication and morphogenesis, but the rate of such changes is cell-dependent.
